# The complete chloroplast genome of *Blepharis ciliaris* (Acanthaceae)

**DOI:** 10.1080/23802359.2019.1681315

**Published:** 2019-10-23

**Authors:** Samaila S. Yaradua, Dhafer A. Alzahrani, Abidina Abba, Enas J. Albokhary, Abubakar Bello

**Affiliations:** a Department of Biological Sciences, King Abdulaziz University, Jeddah, Saudi Arabia;; b Centre for Biodiversity and Conservation, Department of Biology, Umaru Musa Yaradua University, Katsina, Nigeria

**Keywords:** Acanthaceae, *Blepharis ciliaris*, chloroplast genome, phylogeny

## Abstract

*Blepharis ciliaris* is an important medicinal plant and endemic species in Saudi Arabia. This study reported the complete chloroplast genome of *B. ciliaris*, the second to be sequence in non cystolith clade of Acanthaceae. The genome is 149, 717 bp in size and consisted of a pair of inverted repeat (25, 331 bp each) separating the two single copy region, the large single copy (LSC) 82, 057 bp and small single copy SSC 16, 998 bp. The plastome has overall GC content of 38.5% and 112 genes comprising of 79 protein coding genes, 30 tRNA genes and 4 rRNA genes. The phylogenetic relationship analysis showed that B. ciliaris is sister to Aphelandra knappiea. The cp genome reported in this study will useful in genetic diversity and evolutionary studies of the species.

## Introduction

*Blepharis ciliaris* (L.) B. L. Burtt. is a member of the tribe Acantheae (Acanthaceae), the species is known to be distributed in Saudi Arabia, East Africa, East Pakistan and Egypt (Kamal and Abdul 1956). The plant is used as fodder for ruminant animals particularly camel and sheep. The plant seeds (powdered) are used as an antibacterial on boils, wounds and sores. The seed of the plant is being used to treat cough, and has diuretic, aphrodisiac and antibacterial activity (Deshpande 2006). In addition the charcoal from the root is used to improve eye vision and treat sore eyes (Boulos 1981; Tackholm 1974).

Despite the important of complete chloroplast genome in evolutionary studies and taxonomy, only the cp genome of 8 genera has been reported so far out of ca. 4000 species of Acanthaceae, all the 7 genera belong to cystolith clade, only 1 genus belongs to non cystolith clade. In this study, we reported the second chloroplast genome of non cystolith clade of Acanthaceae *B. ciliaris* and constructed the phylogenetic relationship with other Acanthaceae

Plant material was collected from Biological garden, King Abdulaziz University, Jeddah, Saudi Arabia (39°15′ 0.60″E, 21°29′ 22.79″N). Voucher specimen was deposited at the Herbarium of King Abdulaziz University with voucher specimen number KAU22535.Total genomic DNA was extracted from leaves using Qiagen DNA extraction Kit according to manufacturer’s protocol. The genomic DNA was sequenced using Illumina Hiseq 2500 platform (Novogene Technologies, Inc. Beijing, China). Raw data reads were filtered by PRINSEQ lite Ver0.20.4 to get clean reads (5GB). The cp genome was assembled from the high quality clean reads using NOVOplasty2.7.2 with kmer 39 using the cp genome of *Strobilanthes cusia* (NC_037485) as seed and reference. Dual Organellar GenoMe Annotator DOGMA (Wyman et al. 2004) was used to annotate the genes in cp genome followed by manual adjustment using BLAST (https://blast.ncbi.nlm.nih.gov/Blast.cgi). trNAscan-SE2.0 (Lowe and Chan 2016) was used to verified tRNA genes. The complete chloroplast genome sequence of *B. ciliaris* was submitted to GenBank (Accession number MK548576).

The complete chloroplast genome of *B. ciliaris* is 149, 717 bp in length and has a typical circular structure with overall GC and AT content of 38.5% and 61.5% respectively, comprising a pair of Inverted Repeats of 25, 331 bp in length separating the Large Single Copy (LSC) 82, 057 bp and Small Single Copy (SSC) 16, 998 bp with. The genome contains 113 unique genes, comprising of 79 protein coding genes, 30 tRNA genes and 4 rRNA genes. Six protein coding genes (*rpl23, ycf2,ycf15,ndhB,rps7* and *rps12,*), seven tRNA genes (*trnI-CAU, trnL-CAA*, *trnV-GAC*, *trnI-GAU*, *trnA-UGC, trnR-ACG* and *trnN-GUU*), and four rRNA genes *rrn16*, *rrn23*, *rrn4.5* and *rrn5* were duplicated within the Inverted Repeats region. Out of the 113 genes, 12 genes contained one intron and two genes contained 2 intron.

**Figure 1.**
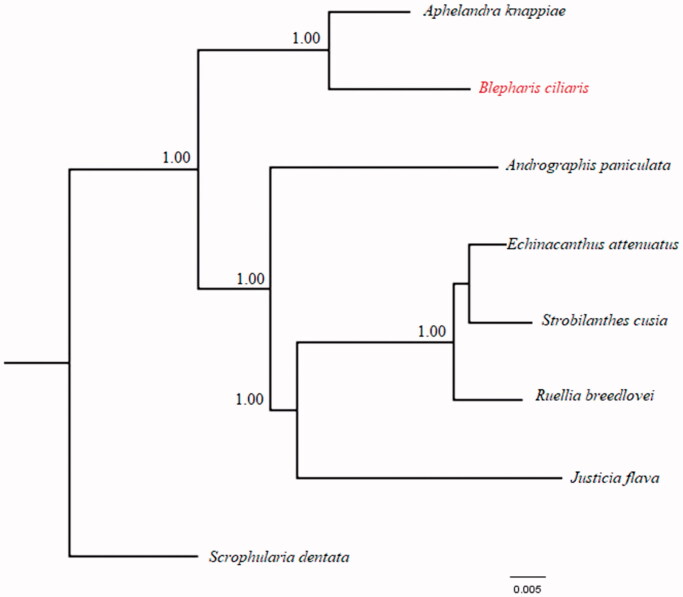
Bayesian Inference (BI) phylogenetic tree of *B. ciliaris* and other Acanthaceae species based on complete chloroplast genome sequence. Numbers in the nodes represent posterior probability (PP) values.

To evaluate the phylogenetic position of *B. ciliaris* in Acanthaceae, cp genome of 7 species of Acanthaceae: *Apheladra knappiea* (MH_909777), *Echinacanthus attenuates*, *strobilanthes cusia* (MG_874806), *Ruellia breedlovei* (KP_300014), *Justicia flava* (MK_548577) and *Andrographis paniculata* (KF_150644) were downloaded from GenBank. The cp genome of *Scrophularia dentata (*KT_428154) was also downloaded from Genbank and used as out group. The downloaded sequences were aligned using MAFFT and analysed using Bayesian Inference with MrBayes version 3.2.6. The result of the phylogenetic analysis showed two distinct clades. The first clade (non cystolith) comprising of *B. ciliaris* and *A. knappiea* and the second clade (cystolith) includes *J. flava*, *S. cusia*, *A. paniculata*, *E. attenuatus* and *R. breedlovei* (Figure 1).
